# TRANSPORTATION-RELATED FACTORS AND THE LIKELIHOOD OF COMPANIONSHIPS AMONG SENIOR CENTER MEMBERS

**DOI:** 10.1093/geroni/igad104.2386

**Published:** 2023-12-21

**Authors:** Rebecca Mauldin, Rupal Parekh, Stephen Mattingly

**Affiliations:** The University of Texas at Arlington, Arlington, Texas, United States; University of Connecticut, Hartford, Connecticut, United States; University of Texas at Arlington, Arlington, Texas, United States

## Abstract

Throughout the United States, a variety of programs focus on social engagement to reduce social isolation and loneliness for older adults. An ideal site for this is the senior center. This research takes place in a senior center serving older Hispanic/Latinx adults in an urban community in the northeast United States. Data were collected 9/21-12/21 from 38 of approximately 42 regular members. Cross-sectional social network analysis data were analyzed using exponential random graph modeling (ERGM) of the likelihood of companionship ties among 42 senior center members. Several transportation related factors were significantly associated with the likelihood of companionship ties. Controlling for demographics and frequency of attendance, having missed going to the senior center due to a lack of transportation was associated with a significantly lower likelihood of a companionship tie (p = .004). Companionships were also less likely for members who were transportation cost-burdened (p = .016). Companionships were less likely for those whose primary mode of transportation was driving compared to those who primarily relied on others for rides (p = .016). Results suggest that transportation experiences are significant factors in the social relationships among senior center members. Finding ways to reduce transportation cost burden and the frequency of missing going to the senior center due to lack of transportation should be considered in interventions to enhance social integration for senior center members. Understanding the role of driving oneself in having companionships for senior center members is an area to explore in future research.

